# Nutritional Status and Indicators of 2-Year Mortality and Re-Hospitalizations: Experience from the Internal Clinic Departments in Tertiary Hospital in Croatia

**DOI:** 10.3390/nu13010068

**Published:** 2020-12-28

**Authors:** Tanja Miličević, Ivana Kolčić, Tina Đogaš, Piero Marin Živković, Maja Radman, Josipa Radić

**Affiliations:** 1 Department of Endocrinology and Diabetology, University Hospital of Split, Spinčićeva 1, 21 000 Split, Croatia; tanja.milicevic2@gmail.com (T.M.); maja.radman1@st.t-com.hr (M.R.); 2 Department of Public Health, University of Split School of Medicine, Šoltanska 2, 21 000 Split, Croatia; ikolcic@mefst.hr; 3 Department of Nephrology and Dialysis, University Hospital of Split, Spinčićeva 1, 21 000 Split, Croatia; tina.dogas@gmail.com; 4 Department of Gastroenterology, University Hospital of Split, Spinčićeva 1, 21 000 Split, Croatia; piero.zivkovic@gmail.com; 5 Department of Internal Medicine, University of Split School of Medicine, Šoltanska 2, 21 000 Split, Croatia

**Keywords:** malnutrition, NRS-2002, internal medicine, elderly, mortality, re-hospitalization, oral nutritional supplement

## Abstract

We aimed to provide insight into nutritional and clinical indicators of malnutrition risk and their influence on two-year mortality and re-hospitalization rate among patients hospitalized in internal clinic departments in the tertiary hospital in Croatia. Initially, data on 346 participants were obtained, while 218 of them where followed-up two years later. At baseline, the majority of participants were old and polymorbid (62.1% suffered from arterial hypertension, 29.5% from cancer, and 29.2% from diabetes). Even apparently presenting with satisfying anthropometric indices, 38.4% of them were at-risk for malnutrition when screened with the Nutritional Risk Screening-2002 (NRS-2002) questionnaire (NRS-2002 ≥ 3). More importantly, only 15.3% of all participants were prescribed an oral nutritional supplement during hospitalization. Those that were at-risk for malnutrition suffered significantly more often from cancer (54.9% vs. 20.6%; *p* < 0.001) and died more often in the follow-up period (42.7% vs. 23.5%; *p* < 0.003). Their anthropometric indices were generally normal and contradictory 46.3% were overweight and obese (body mass index (BMI) > 25 kg/m^2^). Only 36.6% of nutritionally endangered participants used an oral supplement in the follow-up period. NRS-2002 ≥ 3 correlated with anthropometric indices, glomerular filtration rate, age, and length of the initial hospital stay. Unlike other studies, NRS-2002 ≥ 3 was not an independent predictor of mortality and re-hospitalizations; other clinical, rather than nutritional parameters proved to be better predictors. Patients in our hospital are neither adequately nutritionally assessed nor managed. There is an urgent need to develop strategies to prevent, identify, and treat malnutrition in our hospital and post-discharge.

## 1. Introduction

Hospital malnutrition is prevalent but frequently overlooked health condition. This is not surprising as it can derive from a variety of conditions such as starvation, acute or chronic disease, advanced aging, alone or in combination [1]. Depending on the country, selected population, health-care setting, and used diagnostic criteria, international studies report on the hospital malnutrition prevalence rates ranging from 20% to 50% [2]. What matters most is the fact that malnutrition seems to be independently associated with poor hospitalization outcomes, decreased early and late survival, more frequent hospital readmissions, as well as an increased cost of care [3,4,5,6,7]. Taking this into account, the latest clinical guidelines brought by the European Society of Parenteral and Enteral Nutrition (ESPEN) propose that all subjects that come in contact with the health-care system should undergo malnutrition risk screening with a validated screening tool [8]. The Nutritional Risk Screening-2002 (NRS-2002) is a simple, effective, and superior screening tool in identifying the malnutrition risk among acutely hospitalized patients when compared to different screening tools [8]. In subjects identified as being at-risk of malnutrition, detailed nutritional assessment should provide the diagnosis of malnutrition and further nutritional care plan. This appears to be neglected due to the lack of a universal consensus for malnutrition diagnostic criteria and their cut-offs, and this could, even partially, explain high rates of hospital malnutrition despite clear recommendations for its recognition and management [8]. Several studies report on NRS-2002 as a strong, modifiable, and independent predictor of malnutrition associated mortality, length of stay (LOS), and adverse outcomes in different non-intensive care populations (e.g., surgical, internal, chronic obstructive pulmonary disease (COPD), hemodialysis) [9,10,11,12,13]. Nonetheless, many indicators of malnutrition risk (e.g., leucocyte count, serum albumin, C reactive protein (CRP), body mass, comorbidity index, food intake) have also been shown to correlate with clinical outcomes [7,14,15,16,17]. However, even though there are several screening tools for malnutrition risk screening and nutritional assessment available, it seems that there is still no “gold standard” tool that can reliably and independently predict poor-nutrition-related outcomes [14]. It is important to highlight that different studies use different tools which makes it impossible to compare the results.

Nutritional screening and assessment have not been a part of the clinical routine in Croatian hospitals. Unlike some neighboring countries where the national recommendations for malnutrition screening and assessment have been established [18], in Croatia, we are lacking in national guidelines. There are few recommendations regarding nutrition in the elderly [19,20] or malnutrition screening and prevention in specific conditions such as COPD [21], chronic kidney disease (CKD) [22], and preoperative management [23]. Data on the prevalence of hospital malnutrition in Croatian patients is incomplete. Searching the literature, we have found a study evaluating malnutrition risk in patients admitted to internal departments in one tertiary hospital [24], one in the Gastroenterology department [25] and one in children [26]. However, data on long-term malnutrition outcomes in our population is lacking. There is an urgent need in Croatia to increase the health-care system awareness regarding nutrition-related health problems and to promote methods for the identification and treatment of these conditions.

In this study, we aimed to assess the nutritional status and other indicators of nutritional risk and their impact on two-year mortality and hospital readmissions among patients hospitalized in an internal medicine clinic in the second-largest hospital center in Croatia.

## 2. Materials and Methods

### 2.1. Study Design and Setting

This prospective cohort study enrolled adult patients hospitalized in the internal clinic departments in the University Hospital of Split during November 2015. After elaboration of the initial idea and the approval of the Ethics Committee, data acquisition began. A period of one month was defined by agreement, taking into account the daily work responsibilities and the workload of the examiners. Exclusion criteria were critically ill, immobile patients, those with lower limb amputation, patients unable to communicate, and those unwilling to participate in the study. Three educated medical doctors conducted data and measurements on the study participants in the first 48 h of their admittance to the hospital. Two years later, each participant or a family relative (in the case of a deceased participant) with a known telephone number was contacted, and data were collected. Follow-up data collection was completed by the end of 2017.

**Figure 1:**
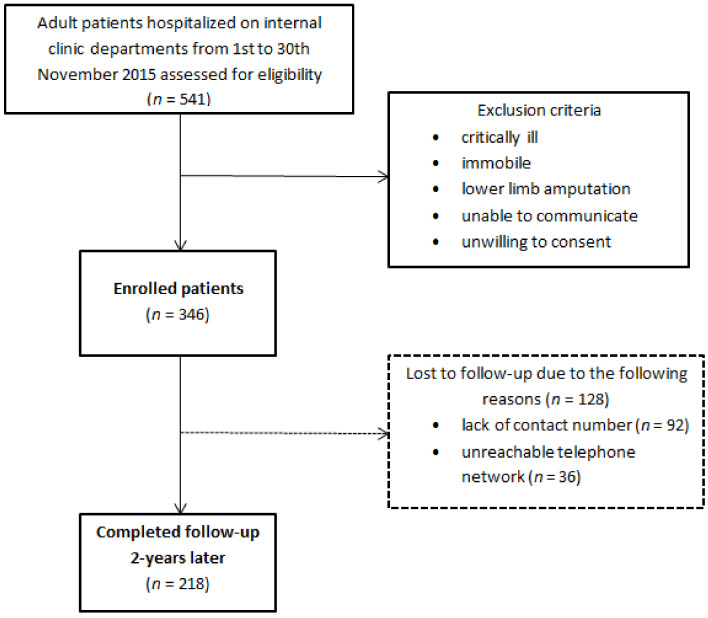
Study design.

Out of a total of 541 hospitalized patients, 346 were enrolled in the study at the beginning, while 218 of them were followed-up two years after the initial hospitalization. A total of 128 subjects have been lost to follow-up, mainly due to the inability to establish a proper telephone visit (lack of contact number or unreachable telephone network) (Figure 1). All participants were informed of the purpose and nature of the study and provided written consent. The study protocol was accepted by the Ethics Committee of the University Hospital of Split (Class 500-03/15-01/39, Number 2181-147-01/06/J.B.-13-2; Class 500-03/17-01/04, Number 2181-147-01/06/M.B.-16-2) and the study was performed following the guidelines of the latest version of the declaration of Helsinki.

### 2.2. Demographic, Anthropometric, Laboratory, and Medical History Assessment

At the beginning of the study data on gender and age were collected. Performed anthropometric measurements included height, weight, body mass index (BMI), waist circumference (WC), Waist-to-Height Ratio (WHtR), forearm circumference, and forearm skin fold. The weight was measured with the participants standing in the orthostatic position, with the arms extended along the body, being barefoot, and wearing light clothes. A stadiometer with a precision of 0.1 cm was used to measure height, with the participants standing barefoot [27]. BMI was calculated as weight in kilograms divided by the square of the height in meters (kg/m^2^) [27]. Standard BMI categories proposed by World Health Organization (WHO) were used [28]. Recently, Global Leadership Initiative on Malnutrition (GLIM) criteria for the diagnosis of malnutrition were adopted, proposing higher BMI cut-offs as a criterion for undernutrition, especially in people older than 70 years [29]. According to them, BMI < 20 kg/m^2^ in people younger than 70 years and BMI < 22 kg/m^2^ in people older than 70 years imply undernutrition. WC was measured using a flexible non-elastic measuring tape. Participants stood with their feet together and arms resting by their sides. WC was taken as the plane between the umbilical scar and the inferior rib border [30]. WC cut-off points for abdominal obesity in metabolic syndrome definition were determined (WC ≥ 94 cm in men and ≥80 cm in women) [31]. WHtR was calculated as waist measurement divided by height measurement in centimeters. The standard cutoff points of 0.5 were used for WHtR [32]. Forearm circumference was measured with the measurer standing behind the participant and locating the mid-upper arm point on the non-dominant arm (midpoint between the lateral tip of the acromion and the most distant point on the olecranon). The non-elastic measuring tape was then placed at the marked midpoint and the circumference of the upper arm was measured to the nearest centimeter [27]. To assess the forearm skin fold, the Accu-Measure Fitness 3000 Body Fat Caliper^®^ was used. The examiner stood behind the participant who was holding hands free to the side of the body. After locating the mid-upper point on the right arm, the examiner grasped the skinfold firmly between the thumb and index finger. The skin fold was lifted 1 cm and recorded with the caliper. A minimum of two measurements was recorded. The acceptable range between repeated measures was 1 mm [27]. If the values varied by more than 1 mm, an additional measurement was taken and the average of the three measurements was used.

Data on accompanied comorbidities (arterial hypertension, diabetes mellitus, active cancer, chronic kidney disease (CKD), liver cirrhosis, inflammatory bowel disease, autoimmune disease) as well as on laboratory findings (creatinine, glucose, and CRP level) were collected from the medical documentation or from the interview with the participant.

Cancer diagnosis included active malignant disease in terms of either solid cancer (e.g., lung, gastrointestinal, breast, kidney, prostate cancer) or hematologic malignancy (lymphoma or leukemia). Participants with benign types of cancers (e.g., skin cancer, except for melanoma) or those who had been cured were not included in this subgroup. The estimated glomerular filtration rate (eGFR) was calculated using the CKD-EPI formula (available at www.niddk.nih.gov).

### 2.3. Nutritional Assessment

Malnutrition risk was assessed with the NRS-2002 screening tool which consists of the evaluation of the nutritional status (scored 0 to 3) and disease severity (scored 0 to 3), with an extra score of 1 for patients older than 70 years. Score ≥ 3 indicated that the participant was at-risk for malnutrition. We also noted whether the participant was adhering to dietary advice or taking any form of nutritional support (vitamin supplement or oral nutritional supplement (ONS)) prior to the initial hospitalization. Data on the length of the initial hospitalization and ONS initiation were recorded.

### 2.4. Follow-Up

The telephone visit was performed two years after the initial hospitalization. We spoke to the participant or a family member in the event of the participant’s death. Data on survival, weight change, newly developed cancer, arterial hypertension, and diabetes mellitus were recorded. From the interview with the participant and available medical records, we also collected information on the number of re-hospitalizations (one, two, three, four, or more) in the follow-up period. We also noted if the participants were taking ONS in the meantime.

### 2.5. Statistical Analysis

Data were summarized as absolute numbers and percentages for categorical variables, and medians and interquartile range (IQR) for numerical variables due to non-normal distribution of the data (tested with Kolmogorov–Smirnov test). To analyze the differences between groups, we used the Chi-square test, Fisher exact test, and the Mann–Whitney U test. The correlation between variables was analyzed using Spearman’s rank correlation test. Finally, we used binary logistic regression in order to identify risk factors for re-hospitalization and death during the follow-up period (in two separate models). Independent variables in each of the two regression models were gender, age, smoking, forearm circumference as the anthropometric indicator, NRS-2002 ≥ 3, CRP concentration, eGFR group at the baseline, ONS use during the follow-up period, and diabetes, cancer, and CKD diagnosis at the baseline. Within the regression model for death outcome, we also included data on re-hospitalization as the predictor. Statistical analysis was performed using SPSS Statistics software v21.0 (IBM, Armonk, NY, USA). Statistically, significance was set at *p* < 0.05.

## 3. Results

Out of 346 initially enrolled participants, 218 were followed-up for 2 years, while 128 participants were lost to follow-up. The baseline characteristics of both groups of participants are shown in Table 1. There was no difference in age, gender composition, and smoking habits between participants with follow-up and those without. Both groups had increased anthropometric indices, without the difference between them, except in forearm skinfold thickness, with followed-up participants having on average higher value (39 mm vs. 28 mm; *p* < 0.001) (Table 1). Participants in the follow-up group had longer initial hospitalization (11 days vs. 8 days; *p* = 0.006), and based on NRS-2002 ≥ 3 criterion, 37.6% of these participants were nutritionally at-risk which was similar to participants without follow-up (40.2%; *p* = 0.640). Regarding the assessed comorbidities in followed-up participants, where participants could have had more than one diagnosis, the most common were arterial hypertension (59.6%), cancer (33.5%), diabetes (29.4%), CKD (26.1%), followed by autoimmune disease (16.5%), liver cirrhosis (4.1%), and inflammatory bowel disease (1.8%). Participants who were lost to follow-up were less frequently diagnosed with cancer and CKD (22.7% and 10.9%, respectively), and they on average had better GFR and lower creatinine (Table 1). The majority of followed-up participants (58.7%) had not received any kind of nutritional support prior to initial hospitalization, and only 21.2% of them were prescribed an ONS during hospitalization. In the lost to follow-up group, 78.9% of participants had not received any nutritional support prior to hospitalization, while only 5.5% of them were prescribed an ONS during hospitalization (Table 1).

Stratification of followed-up participants according to the NRS-2002 score revealed that those participants who were initially at-risk for malnutrition (NRS-2002 ≥ 3) were significantly older (*p* < 0.001), dominantly above 65 years of age (72% of participants vs. 43.4% in participants with NRS-2002 < 3; *p* < 0.001), non-smokers (*p* = 0.030), and had longer initial hospital stay (*p* < 0.001) at the baseline (Table 2). They were on average thinner, with 6.1% of participants being underweight (BMI < 18.5 kg/m^2^), 47.6% having normal BMI (BMI 18.5–24.9 kg/m^2^), 37.8% were overweight (BMI 25–29.9 kg/m^2^), while more than 8.5% were obese (BMI > 30 kg/m^2^). When BMI was stratified according to GLIM criteria [29], 7 participants younger than 70 years and 14 participants older than 70 years fulfilled malnutrition criteria. There was substantially higher percentage of undernourished patients (according to GLIM criteria) in the NRS-2002 ≥3 group (20.7% vs. 2.9%, *p* < 0.001). Their average waist circumference was 99.5 cm (IQR 18.0), forearm circumference 27 cm (IQR 5.0), and forearm skin fold 35 mm (IQR 11.0). Nutritionally endangered participants had significantly lower values of waist circumference (99.5 cm vs. 104.3 cm, *p* < 0.001) and forearm circumference (27 cm vs. 31 cm, *p* < 0.001) when compared to those participants who were not at-risk for malnutrition.

Considering the aforementioned comorbidities, cancer was significantly more common among nutritionally endangered participants (54.9% vs. 20.6%; *p* < 0.001), followed by arterial hypertension (68.3% vs. 54.4%; *p* = 0.043), while none of them suffered from liver cirrhosis. Even though there was no statistically significant difference in CKD prevalence between these two groups, there was a significant difference in GFR (*p* = 0.006). Only 9.8% of participants with NRS-2002 ≥ 3 had sufficient renal function (GFR ≥ 90 mL/min), unlike 27.8% of participants with NRS-2002 < 3 (*p* = 0.008), and 15.9% of participants who were nutritionally at-risk had GFR in the range from 15 to 29.9 mL/min, and in additional 12.2% participants GFR was <15 mL/min. There was no difference in nutritional support prior to and during hospitalization between these two groups.

In the follow-up period, participants that were initially at-risk of malnutrition more frequently used ONS (36.6% vs. 23.5%; *p* = 0.005), and their weight increased on average by 4.9% (IQR 15.3%). Participants who were not initially at-risk for malnutrition were more often diagnosed with cancer during the follow-up period (12.5% vs. 3.7%; *p* = 0.028). There was no difference considering the re-hospitalization rate between these two groups, but high percentage of participants in both groups were re-hospitalized within 2 years of follow-up (70.4% in those with NRS-2002 ≥ 3 and 59.6% in those with NRS-2002 < 3). Finally, participants who were initially at-risk for malnutrition experienced a higher share of death outcomes (42.7% vs. 23.5%; *p* = 0.003) (Table 2).

NRS-2002 was correlated negatively with BMI, waist circumference, forearm circumference, and GFR rate, while a positive correlation was recorded with age and the length of initial hospital stay (all *p* < 0.001) (Table 3). The strongest correlation was recorded between NRS-2002 score and forearm circumference (r = -0.374; *p* < 0.001), unlike the measures of central obesity, such as waist-to-height ratio (r = −0.101; *p* = 0.140) and waist circumference (r = −0.188; *p* = 0.005).

Predictors of re-hospitalization and death outcomes during the follow-up period are shown in Table 4. CRP concentration (OR = 1.01, 95% CI 1.00–1.02; *p* = 0.032), GFR < 15mL/min (OR = 12.49, 95% CI 1.22–127.61; *p* = 0.033), and use of ONS (OR = 2.70, 95% CI 1.11–6.54; *p* = 0.028) were significant risk factors for re-hospitalization, while NRS-2002 ≥ 3 was not a significant predictor in fully adjusted regression model. Significant predictors of death outcome during the follow-up period were forearm circumference (OR = 0.87, 95% CI 0.78–0.96; *p* = 0.008), use of ONS (OR = 4.24, 95% CI 1.80–9.97; *p* = 0.001), diabetes (OR = 3.74, 95% CI 1.54–9.06; *p* = 0.003), and cancer diagnosis (OR = 5.85, 95% CI 2.23–15.33; *p* < 0.001), while NRS-2002 ≥ 3 was not a significant predictor in the fully adjusted regression model.

## 4. Discussion

Few studies have assessed nutritional status of adult patients hospitalized in internal clinic departments in Croatian hospitals [24,25]. However, since there are no long-term outcome studies, our study is of great importance. We provide insight into the nutritional status, assessment, and for the first time, long-term outcomes of patients acutely hospitalized in internal clinic departments in the Croatian tertiary hospital.

As in other internal clinics, our patients were generally old and polymorbid. While almost 30% of them suffered from cancer, the majority appeared to be well to over-nourished when the anthropometric parameters were assessed. Considering the increasing share of obesity, it becomes challenging to identify malnutrition in the overweight population and in those needing nutritional intervention. When all the participants were evaluated with NRS-2002 screening tool, almost 40% of them were actually at-risk for malnutrition. Moreover, every third participant with NRS-2002 ≥ 3 was overweight, while 6% of them were obese. BMI cut-off values (according to GLIM criteria) [29], unlike WHO criteria [28], appeared to be more sensitive in detecting malnourished elderly participants, and these values matched NRS-2002 status well. Unlike standard BMI cut-offs, which seem to be a less reliable indicator due to increased obesity in our subjects, other anthropometric indicators suggestive of increased nutritional risk were lower waist and particularly lower forearm circumference. This discrepancy between body proportions presented as BMI and the real nutritional status is probably the reason that only minority of our participants had been using ONS before and during hospitalization (13.3% and 15.3%, respectively). It is even more worrying that only 36.6% of those at-risk for malnutrition used ONS in the follow-up period. Interestingly, participants that were lost to follow up received ONS before and during hospitalization less frequently, even being at the same nutritional risk as followed-up participants. One of the reasons could be the lower prevalence of cancer in this group, but also the lack of general malnutrition awareness and screening certainly contribute. Our results coincide with other studies reporting on a high rate of in-hospital malnutrition, its non-recognition, and inadequate management [33,34].

Participants that were at-risk for malnutrition suffered significantly more often from cancer (almost 55% of them) which is both expected and worrying in this patient population. Nutritional risk assessed as NRS-2002 ≥ 3 among cancer patients varies from 30% to 68% depending on the cancer type, stage, and type of medical care provided (inpatient/outpatient) [35,36].

In our study, every third nutritionally endangered participant suffered from CKD. In the study by Sorensen et al. [37] malnutrition was detected in 29% CKD patients, while Borek et al. [38] reported on 39% of malnourished CKD patients with the nutritional status deteriorating with decreasing GFR. Even though there was no significant difference in CKD history among NRS-2002 groups, we must emphasize a significantly lower percentage of nutritionally endangered participants having eGFR > 90 mL/min, based on creatinine value on admission. This association could be explained by the fact that acute (transient) kidney insufficiency in acute illness causes appetite loss, potentiates weight loss, and leads to malnutrition risk.

Unlike other studies that found NRS-2002 ≥ 3 to be a strong and independent prognostic factor of early and late mortality [7,9], and re-hospitalization rate [9], in our study NRS-2002 was not a significant factor of either two-year mortality or re-hospitalization rate. It was, however, associated with a longer initial hospital stay. It remains unclear if the aforementioned association between NRS status, mortality, and re-hospitalization rate can be explained by other disease-related factors, or whether the nutritional support may influence the connection between nutritional status and outcome.

Some systematic reviews suggested that high-protein ONS significantly reduced re-hospitalizations [39] and mortality [40] compared with controls, while other systematic reviews and meta-analyses failed to show consistent results [41,42]. Recent randomized double-blind clinical-trial with ONS failed to prove a lower 90-day readmission rate, but 90-day mortality lowered and nutritional status improved [43]. In our study, the use of ONS was associated with a higher mortality and re-hospitalization rate. Such results are probably due to late malnutrition recognition, delayed ONS initiation, and a large share of cancer patients indicating poorer prognosis and outcome.

In our study, the best nutritional predictor of mortality was forearm circumference, while other clinical, rather than nutritional parameters predicted re-hospitalization rate (CRP level, eGFR < 15 mL/min, and ONS use) and two-year mortality (cancer, diabetes, ONS use).

This study has some limitations. Unfortunately, after initial screening we had to exclude 195 patients since they were either unable/unwilling to consent or unable to perform anthropometric measurements. Taking into account that certain proportion of these patients was critically ill, unconscious, and immobile or with amputated limbs, we strongly believe that even higher proportion of nutritionally endangered participants would be detected among them. Therefore, our results do not apply to such patient population. Our results are based only on the admission NRS-2002 score with no longitudinal follow-up. We did not compare NRS-2002 to other nutritional status assessment tools or implemented any appetite questionnaires. This study involved older internal medical patients who generally suffer from multiple comorbidities, so our results may not be applicable to younger subspecialty patients with single organ system involvement. Moreover, it is well known that older persons show varying degree of cognitive and psychological impairment and that malnutrition and adverse outcomes are more frequently observed in participants presenting with these disturbances [44]. Unfortunately, these clinical aspects were not evaluated in our study. Also, there was a big drop-out of the participants in the follow-up period mainly because of the lack of adequate/reachable contact numbers which could have affected generalizability of our results. However, it is worth noting that almost 2 million people gravitate to our hospital center. So, despite the single-center nature of our study and small number of participants, the main strength lies in the representative patient sample within a prospective observational setting. Further studies with larger sample size are desirable.

## 5. Conclusions

In conclusion, the significant number of patients hospitalized in internal clinic departments is at-risk for malnutrition, despite presenting with apparently satisfying anthropometric indicators. These patients are not adequately nutritionally evaluated and managed. NRS-2002 ≥ 3 is not a good predictor of the two-year mortality and re-hospitalization rate in our patient population. Better predictors of re-hospitalization rate and two-year mortality are different clinical parameters rather than nutritional indicators. There is an urgent need to develop strategies to prevent, identify, and treat malnutrition in our hospital and post-discharge.

## Figures and Tables

**Table 1:** Baseline characteristics of subjects.

	All Subjects *n* = 346	Follow-Up *n* = 218	Lost to Follow-Up *n* = 128	*p*
Socio-demographic characteristics				
Age (years); median (IQR)	67.0 (19.0)	66.0 (18.0)	68.0 (21.0)	0.080 †
Gender; *n* (%)				
Female	157 (45.4)	105 (48.2)	52 (40.6)	0.174 *
Male	189 (54.6)	113 (51.8)	76 (59.4)	
Habits; *n* (%)				
Smoking	72 (20.8)	43 (19.7)	29 (22.8)	0.493 *
Anthropometric indices; median (IQR)				
BMI (kg/m^2^)	26.3 (7.0)	26.4 (7.0)	26.3 (6.0)	0.515 †
Waist circumference (cm)	103.0 (18.0)	102.9 (18.0)	103.5 (18.0)	0.963 †
WHtR	0.59 (0.10)	0.59 (0.10)	0.60 (0.11)	0.557 †
Forearm circumference (cm)	29.5 (6.5)	29.8 (6.5)	29.5 (7.0)	0.607 †
Forearm skinfold (mm)	35.0 (12.0)	39.0 (15.0)	28.0 (14.0)	<0.001 †
Chronic disease; *n* (%)				
Hypertension	215 (62.1)	130 (59.6)	85 (66.4)	0.210 *
Diabetes	101 (29.2)	64 (29.4)	37 (28.9)	0.929 *
Cancer	102 (29.5)	73 (33.5)	29 (22.7)	0.033 *
CKD	71 (20.5)	57 (26.1)	14 (10.9)	0.001 *
IBD	10 (2.9)	4 (1.8)	6 (4.7)	0.117 **
Cirrhosis	17 (4.9)	9 (4.1)	8 (6.3)	0.378 *
Autoimmune disease	51 (14.7)	36 (16.5)	15 (11.7)	0.224 *
Biochemistry; median (IQR)				
CRP (mg/L)	11.7 (34.1)	11.6 (31.8)	11.8 (46.1)	0.977 †
Glucose (mmol/L)	6.4 (3.0)	6.4 (2.8)	6.3 (3.4)	0.790 †
Creatinine (μmol/L)	91.0 (55.0)	95.0 (79.0)	79.5 (38.0)	<0.001 †
eGFR (mL/min)	69.1 (49.1)	66.2 (51.5)	76.7 (48.1)	0.001 †
Nutritional risk assessment; *n* (%)				
NRS-2002 ≥ 3	133 (38.4)	82 (37.6)	51 (40.2)	0.640 *
Nutritional support prior to hospitalization; *n* (%)				
None	229 (66.2)	128 (58.7)	101 (78.9)	<0.001 **
Nutritional advice	7 (2.0)	6 (2.8)	1 (0.8)	
Vitamin supplement	60 (17.3)	47 (21.6)	13 (10.2)	
ONS	46 (13.3)	37 (17.0)	9 (7.0)	
Nutritional support included during hospitalization; *n* (%)				
ONS	53 (15.3)	46 (21.1)	7 (5.5)	<0.001 *
Hospitalization length; median (IQR)				
Initial hospitalization (days)	11.0 (7.0)	11.0 (8.0)	8.0 (7.0)	0.006 †

IQR—interquartile range; BMI—body mass index; WHtR—Waist-to-Height Ratio; NRS-2002—Nutritional Risk Screening-2002; CKD—chronic kidney disease; IBD—inflammatory bowel disease; CRP—C reactive protein; eGFR—estimated glomerular filtration rate; ONS—oral nutritional supplement; * chi-square test, † Mann-Whitney U test, ** Fisher exact test.

**Table 2:** Characteristics of subjects according to the NRS-2002 score in the followed-up sample.

	NRS-2002 < 3 *n* = 136	NRS-2002 ≥ 3 *n* = 82	*p*
Baseline			
Socio-demographic characteristics			
Age (years); median (IQR)	62.0 (19.0)	73.0 (16.0)	<0.001 †
Age groups; *n* (%)			<0.001 *
<65 years	77 (56.6)	23 (28.0)	
≥65 years	59 (43.4)	59 (72.0)	
Gender; *n* (%)			0.208 *
Female	61 (44.9)	44 (53.7)	
Male	75 (55.1)	38 (46.3)	
Habits; *n* (%)			
Smoking	33 (24.3)	10 (12.2)	0.030 *
Anthropometric indices			
BMI (kg/m^2^); median (IQR)	28.0 (7.0)	24.8 (7.0)	<0.001 †
BMI category (kg/m^2^); *n* (%)			<0.001 **
<18.5	0 (0.0)	5 (6.1)	
18.5–24.9	42 (30.9)	39 (47.6)	
25.0–29.9	49 (36.0)	31 (37.8)	
30.0–34.9	32 (23.5)	6 (7.3)	
35.0–39.9	10 (7.4)	1 (1.2)	
≥40	3 (2.2)	0 (0.0)	
Undernutrition according to GLIM criteria ^§^; *n* (%)			<0.001 *
Yes	4 (2.9)	17 (20.7)	
No	132 (97.1)	65 (79.3)	
Waist circumference (cm); median (IQR)	104.3 (17.0)	99.5 (18.0)	<0.001 †
WHtR; median (IQR)	0.59 (0.12)	0.59 (0.11)	0.026 †
WHtR ≥0.5; *n* (%)	128 (94.1)	71 (86.6)	0.056 *
Forearm circumference (cm); median (IQR)	31.0 (6.0)	27.0 (5.0)	<0.001 †
Forearm skinfold (mm); median (IQR)	40.0 (14.0)	35.0 (11.0)	0.026 †
Chronic disease; *n* (%)			
Hypertension	74 (54.4)	56 (68.3)	0.043 *
Diabetes	42 (30.9)	22 (26.8)	0.524 *
Cancer	28 (20.6)	45 (54.9)	<0.001 *
CKD	31 (22.8)	26 (31.7)	0.147 *
IBD	2 (1.5)	2 (2.4)	0.633 **
Cirrhosis	9 (6.6)	0 (0.0)	0.015 **
Autoimmune disease	22 (16.2)	14 (17.1)	0.863 *
Biochemistry; median (IQR)			
CRP (mg/L)	9.7 (33.7)	14.6 (27.6)	0.440 †
Glucose (mmol/L)	6.4 (3.0)	6.3 (2.4)	0.779 †
Creatinine (μmol/L)	93.0 (57.0)	104.0 (116.0)	0.208 †
eGFR (mL/min)	72.2 (49.8)	56.7 (51.1)	0.006 †
eGFR category; *n* (%)			0.008 *
<15.0	17 (12.8)	10 (12.2)	
15.0–29.9	8 (6.0)	13 (15.9)	
30.0–59.9	30 (22.6)	20 (24.4)	
60.0–89.9	41 (30.8)	31 (37.8)	
≥90.0	37 (27.8)	8 (9.8)	
Nutritional support prior to hospitalization; *n* (%)			0.065 **
None	80 (58.8)	48 (58.5)	
Nutritional advice	4 (2.9)	2 (2.4)	
Vitamin supplement	35 (25.7)	12 (14.6)	
ONS	17 (12.5)	20 (24.4)	
Nutritional support included during hospitalization; *n* (%)			
ONS	25 (18.4)	21 (25.6)	0.205 *
Hospitalization length; median (IQR)			
Initial hospitalization (days)	10.0 (7.0)	14.0 (9.0)	0.001 †
Follow-up period			
Percent weight change (%); median (IQR)	+0.06 (7.1)	+4.9 (15.3)	0.072 †
Newly diagnosed chronic disease; *n* (%)			
Cancer	17 (12.5)	3 (3.7)	0.028 *
Hypertension	4 (2.9)	1 (1.2)	0.652 **
Diabetes	3 (2.2)	0 (0.0)	0.293 **
Re-hospitalizations; *n* (%)			
All re-hospitalizations	81 (59.3)	57 (70.4)	0.109 *
One re-hospitalization	32 (23.7)	25 (30.9)	
Two re-hospitalizations	19 (14.1)	12 (14.8)	
Three re-hospitalizations	9 (6.7)	7 (8.7)	
Four or more re-hospitalizations	20 (14.8)	13 (16.0)	
None	55 (40.7)	24 (29.6)	0.553 *
Nutritional support; *n* (%)			
ONS use	26 (19.3)	30 (36.6)	0.005 *
Adverse outcome; *n* (%)			
Deceased	32 (23.5)	35 (42.7)	0.003 *

IQR—interquartile range; BMI—body mass index; WHtR—Waist-to-Height Ratio; NRS-2002—Nutritional Risk Screening-2002; CKD—chronic kidney disease; IBD—inflammatory bowel disease; CRP—C reactive protein; eGFR—estimated glomerular filtration rate; ONS—oral nutritional supplement; * chi-square test, † Mann-Whitney U test, ** Fisher exact test, ^§^ GLIM criteria for the diagnosis of malnutrition based on low BMI: <20 kg/m^2^ if <70 years, and/or <22 kg/m^2^ if ≥70 years old.

**Table 3:** Correlation between NRS-2002 score and anthropometric indicators and other important clinical parameters, *n* = 218 (data are presented as Spearman’s rho (*p* value)).

	Age	Hospitalization Duration	BMI	Waist circumference	WHtR	Forearm Circumference	Forearm Skinfold	CRP	eGFR
NRS-2002 score	0.471 (<0.001 *)	0.249 (<0.001 *)	−0.308 (<0.001 *)	−0.188 (0.005)	−0.101 (0.140)	−0.374 (<0.001 *)	−0.112 (0.099)	0.094 (0.196)	−0.256 (<0.001 *)
Age		0.088 (0.196)	0.020 (0.770)	0.207 (0.002)	0.320 (<0.001 *)	−0.073 (0.284)	0.069 (0.307)	0.093 (0.198)	−0.499 (<0.001 *)
Hospitalization duration			−0.023 (0.740)	0.012 (0.863)	0.048 (0.482)	−0.121 (0.074)	0.009 (0.895)	0.194 (0.007)	−0.084 (0.222)

NRS-2002—Nutritional Risk Screening-2002; BMI—body mass index; WHtR—Waist-to-Height-Ratio; CRP—C reactive protein; eGFR—estimated glomerular filtration rate. Note: a Bonferroni correction was applied for the correlation analyses in this table with 24 separate *p* values, which sets the significance level at *p* < 0.002, and symbol * denotes significant results after Bonferroni correction.

**Table 4:** Risk factors for death outcome and re-hospitalization during follow-up, *n* = 218 (binary logistic regression).

	Re-Hospitalization OR (95% CI); *p*	Death OR (95% CI); *p*
Gender (women are referent group)	0.51 (0.26–1.00); 0.050	0.81 (0.37–1.79); 0.603
Age (<65 yrs is referent group)	1.56 (0.71–3.46); 0.269	1.67 (0.66–4.25); 0.280
Smoking (non-smokers are referent group)	1.18 (0.49–2.83); 0.710	0.50 (0.16–1.61); 0.247
Forearm circumference	1.00 (0.97–1.03); 0.844	0.87 (0.78–0.96); 0.008
NRS-2002 ≥ 3	1.57 (0.70–3.53); 0.272	0.51 (0.19–1.32); 0.162
CRP	1.01 (1.00–1.02); 0.032	1.00 (0.99–1.01); 0.372
eGFR (≥90.0 is referent)		
<15.0	12.49 (1.22–127.61); 0.033	1.30 (0.25–6.89); 0.755
15.0–29.9	0.60 (0.14–2.59); 0.495	1.02 (0.20–5.29); 0.977
30.0–59.9	1.06 (0.37–3.08); 0.910	0.55 (0.15–2.01); 0.361
60.0–89.9	1.26 (0.50–3.21); 0.624	0.51 (0.15–1.73); 0.279
ONS during follow-up (didn’t take are referent group)	2.70 (1.11–6.54); 0.028	4.24 (1.80–9.97); 0.001
Diabetes (no is referent group)	1.78 (0.81–3.90); 0.153	3.74 (1.54–9.06); 0.003
Cancer (no is referent group)	0.69 (0.30–1.60); 0.391	5.85 (2.23–15.33); <0.001
CKD (no is referent group)	1.49 (0.50–4.39); 0.474	1.76 (0.52–5.99); 0.366
Re-hospitalized (no is referent group)	-	0.74 (0.30–1.83); 0.509

All variables shown refer to the baseline, except the use of ONS that has been collected during follow-up. OR—odds ratio; CI—confidence interval; NRS-2002—Nutritional Risk Screening-2002; CRP—C reactive protein; eGFR—estimated glomerular filtration rate; ONS—oral nutritional supplement; CKD—chronic kidney disease.

## Data Availability

Patient level data are freely available from the last author at josiparadic1973@gmail.com. There is no personal identification risk within this anonymized raw data, which is available after notification and authorization of the competent authorities.
